# CD133 inhibition via autophagic degradation in pemetrexed-resistant lung cancer cells by GMI, a fungal immunomodulatory protein from Ganoderma microsporum

**DOI:** 10.1038/s41416-020-0885-8

**Published:** 2020-05-25

**Authors:** I-Lun Hsin, Ling-Yen Chiu, Chu-Chyn Ou, Wen-Jun Wu, Gwo-Tarng Sheu, Jiunn-Liang Ko

**Affiliations:** 1 Institute of Medicine, Chung Shan Medical University, Taichung, 40201 Taiwan; 2 Department of Medical Research, Ditmanson Medical Foundation Chia-Yi Christian Hospital, Chiayi, 60002 Taiwan; 3 School of Nutrition, Chung Shan Medical University, Taichung, 40201 Taiwan; 4 Division of Medical Oncology, Department of Internal Medicine, Chung Shan Medical University Hospital, Taichung, Taiwan; 5 School of Medicine, Chung Shan Medical University, Taichung, 40201 Taiwan

**Keywords:** Drug development, Cancer therapeutic resistance, Lysosomes, Macroautophagy

## Abstract

**Background:**

Adaptive drug resistance is an unfavourable prognostic factor in cancer therapy. Pemetrexed-resistant lung cancer cells possess high-metastatic ability via ERK–ZEB1 pathway-activated epithelial–mesenchymal transition. GMI is a fungal immunomodulatory protein that suppresses the survival of several cancer cells.

**Methods:**

Cell viability was analysed by MTT, clonogenic, tumour spheroid, and cancer stem cell sphere assays. Western blot assay was performed to detect the protein expression. Chemical inhibitors and ATG5 shRNA were used to inhibit autophagy. Tumour growth was investigated using xenograft mouse model.

**Results:**

GMI decreased the viability with short- and long-term effects and induced autophagy but not apoptosis in A549/A400 cells. GMI downregulated the expression levels of CD133, CD44, NANOG and OCT4. GMI induces the protein degradation of CD133 via autophagy. CD133 silencing decreased the survival and proliferation of A549/A400 cells. GMI suppressed the growth and CD133 expression of A549/A400 xenograft tumour.

**Conclusions:**

This study is the first to reveal the novel function of GMI in eliciting cytotoxic effect and inhibiting CD133 expression in pemetrexed-resistant lung cancer cells via autophagy. Our finding provides evidence that CD133 is a potential target for cancer therapy.

## Background

Autophagy is a cellular recycling mechanism that plays an important role in protein and organelle turnover.^1^ Endoplasmic reticulum (ER) chaperones, proteasome, and autophagy form the protein quality control system.^2^ During autophagy, aggregate-prone proteins would be polyubiquitinated and engulfed in autophagosomes. Autophagosomes dock lysosomes and then fuse as autolysosomes to degrade the engulfed cargos by lysosomal enzymes.^3^ An appropriate level of autophagy protects cells from stress, but a high level of autophagy triggers autophagic cell death.^4^ Autophagy-mediated cell death is a new strategy for anti-cancer drug development.

Cancer stem cells are a small subpopulation of tumour cells that possess similar characteristics to normal stem cells.^5,6^ Cancer cells with high stemness have high proliferative, metastatic, and drug-resistance capabilities.^7^ Different cancer stem cells express different CD antigens.^7^ CD antigens used as markers of lung cancer stem cell include CD44, CD90, CD117, CD133, and CD166.^8^ Different from other CD antigens of lung cancer stem cell markers, the complete function of CD133 remains unclear to date. Recently, a review paper has summarised the potential pro-survival and anti-apoptosis mechanisms of CD133 in cancer stem cells.^9^ Chen et al.^10^ demonstrated that CD133 induces EMT, stemness properties, and tumorigenicity by activating the Src signalling pathway in head and neck cancer cells. These studies indicated that CD133 is a feasible target for cancer therapy.

Pemetrexed, a folate antagonist, suppresses at least three enzymes participating in folate metabolism and DNA synthesis, including dihydrofolate reductase (DHFR), glycinamide ribonucleotide formyltransferase (GRAFT), and thymidylate synthase (TS).^11^ Pemetrexed has been considered as the first-line therapy for non-small-cell lung cancer.^12–14^ Increased expression of TS is the key factor in adaptive pemetrexed resistance.^15,16^ Pemetrexed resistance does not lead to multiple-drug resistance.^15^ However, the metastatic ability of pemetrexed-resistant lung cancer cells is increased via ERK/ZEB1 cascade-mediated epithelial–mesenchymal transition (EMT).^15^

GMI, a fungal immunomodulatory protein from *Ganoderma microsporum*, possesses multi-faced anti-tumour ability. GMI induces autophagic cell death in lung cancer cells via calcium/p53- and Akt/mTOR-dependent signalling pathways.^17,18^ GMI inhibits the survival of multidrug-resistant lung cancer cells via pro-death autophagy.^19^ GMI enhances cisplatin-activated apoptosis in lung cancer cells via abundant autophagosome accumulation stress (AAA stress).^20^ β-catenin inhibition by GMI triggers apoptosis in lung cancer cells bearing either wild‐type or mutated EGFR.^21^ The inhibitory effect of GMI on the IL-6/Stat3 signalling pathway abolishes the stemness and cisplatin resistance of oral carcinoma stem cells.^22^ A recent study has found that GMI enhances 5-FU-induced cytotoxicity in oral cancer cells.^23^

In this study, we found that GMI inhibits the cell viability and sphere formation of pemetrexed-resistant lung cancer cells. GMI decreased the expression levels of CD133, CD44, and stem cell transcription factors NANOG and OCT4. CD133 inhibition was dependent on autophagic degradation elicited by GMI and inhibited the cell survival and proliferation of pemetrexed-resistant lung cancer cells. Oral administration of GMI suppressed the xenograft tumour growth of pemetrexed-resistant lung cancer cells in nude mice. This study is the first to reveal the novel function of GMI in suppressing the CD133 expression and survival of pemetrexed-resistant lung cancer cells.

## Methods

### Cells, chemicals, and GMI

As previously described, A549/A400 cells were cultured and grown in Dulbecco’s modified Eagle’s medium (DMEM) (GIBCO, 12100-046) supplemented with 10% foetal bovine serum (HyClone, SH30070.03) at 37 °C in a humidified atmosphere of 5% CO_2_. A549/A400 cells were generated and provided by Gwo-Tarng Sheu.^15^ As previously described, the pemetrexed-resistant subline A549/A400 was established from parental A549 cells in a stepwise manner by exposure to increasing concentrations of pemetrexed. Briefly, A549 cells in low cellular density were seeded onto 10-cm Petri dish and treated with 50 nM pemetrexed until the surviving cells grew to an obvious colony. The selected colony was amplified in the presence of pemetrexed until confluence before the drug dose was increased in multiples of two for the next round of selection. As previously described, 3-Methyladenine (3-MA) (M 9281), chloroquine diphosphate salt (C 6628), and MG132 (474790) were purchased from Sigma (St. Louis, MO, USA). As previously described, GMI was provided by Mycomagic Biotechnology Co, Ltd. (Taipei, Taiwan), and GMI was generated and extracted as previously described.^24^

### MTT assay

A549/A400 cells (2 × 10^3^ cells/well) suspended in a 100 μL culture medium were seeded into a 96-well plate. Following treatment with GMI for 48 h, the medium was removed, and 100 μL of fresh medium containing 0.5 mg/mL MTT (Sigma, M 2128) was added to the wells. MTT assay was performed as previously described.^17^

### 3D cancer spheroid formation assay

A549/A400 cells (1 × 10^3^ cells) were seeded into a well of ultra-low attachment 96-well plate (Corning Inc., Corning, NY, USA) containing 200 μL of culture medium. After incubation for 96 h, fresh medium containing GMI was added to the well. The spheroids were incubated for another 7 days and observed under inverted light microscopy. The volume of the spheroids was calculated by the formula 0.5 × larger diameter (mm) × small diameter (mm)^2^.^25^

### Cancer stem cell sphere formation assay

A549/A400 cells (5 × 10^3^ cells) suspended in a 2 mL sphere formation medium [DMEM/F12 medium containing 20 ng/mL basic fibroblast growth factor (PeproTech Asia, Rehovot, Israel), 20 ng/mL epidermal growth factor (PeproTech Asia, Rehovot, Israel), 5 μg/mL insulin (91077C, Sigma, St. Louis, MO, USA), 4 μg/mL heparin (H3194, Sigma, St. Louis, MO, USA), 1 µg/mL Hydrocortisone (H0888, Sigma, St. Louis, MO, USA), 0.4% bovine serum albumin (15260037, Invitrogen, Paisley, UK), and 1% methylcellulose (M0512, Sigma, St. Louis, MO, USA)] with or without GMI were seeded into a well of ultra-low attachment six-well plate (Corning Inc., Corning, NY, USA). A 500 μL fresh sphere formation medium was added to the well every 3 days. The cells were incubated for 14 days, and the spheres were observed via inverted light microscopy.

### Flow cytometry

A549/A400 cells (2 × 10^5^ cells) were seeded in a 60 mm dish containing 4 mL of culture medium. After 16 h incubation, the medium was removed and 4 mL of fresh medium containing GMI (0, 0.3, and 0.6 μM) was added to the dish. After treatment for 48 h, the cells were used to investigate apoptosis and acidic vesicular organelles (AVOs) development. Apoptosis was analysed using Annexin V-FITC Apoptosis Detection Kit (556 547, BD Biosciences, California), and acridine orange (A 6014, Sigma, St. Louis, Missouri) was used to analyse the apoptotic cell death and AVO development. The complete protocol for both analyses has been described elsewhere.^17,26^

### Autophagy detection assay

CYTO-ID® Autophagy detection kit 2.0 (ENZ-KIT175-0200, Enzo Life Sciences, Farmingdale, NY, USA) was used to investigate the formation of autophagosomes. A549/A400 cells were seeded in a 96-well dish at a density of 2 × 10^3^ cells per well and then incubated for 16 h. The cells were treated with GMI (0, 0.3, and 0.6 μM) for 48 h, stained with Cyto-ID Green Detection Reagent and Hoechst 33342 Nuclear Stain for 20 min, and then investigated under a fluorescence microscope (ZEISS-Axio Observer Z1, HAL 100 microscope, ZEISS, Germany). The fluorescence strengths in the stained cells were analysed using Gemini EM Fluorescence Microplate Readers.

### Western blot assay

Anti-CD133 (#5860, Cell Signaling, Danvers, MA, USA), anti-CD44 (#3570, Cell Signaling, Danvers, MA, USA), anti-NANOG (#4903, Cell Signaling, Danvers, MA, USA), anti-OCT4 (ab109183, abcam, Cambridge, UK), anti-LC3B (#3868, Cell Signaling, Danvers, MA, USA), anti-ATG5 (#12994, Cell Signaling, Danvers, MA, USA), and anti-β-actin (AC-40, Sigma, St. Louis, Missouri) were used to detect the protein expression levels of CD133, CD44, NANOG, OCT4, LC3B, ATG5, and β-actin. The complete protocol for Western blot assay has been described in a previous publication.^17^

### Immunocytochemistry

A549/A400 cells were seeded onto coverslips in 60 mm plates at a density of 2 × 10^5^ cells/well and then incubated for 16 h. After GMI treatment for 72 h, the cells were fixed in 4% paraformaldehyde for 10 min, permeabilised with 1% Triton X-100 for 10 min, and then blocked in 1% Bovine Serum Albumin (BSA) in Phosphate Buffered Saline Tween-20 (PBST) for 1 h at room temperature. The cells were hybridised with anti-CD133 (#5860, Cell Signaling, Danvers, MA, USA) antibodies overnight in blocking buffer at 4 °C. Subsequently, the cells were exposed to donkey anti-rabbit-FITC antibody at room temperature for 1 h. The cells were stained with 4′,6-diamidino-2-phenylindole for 5 min at room temperature and observed under a fluorescence microscope (ZEISS-Axio Observer Z1, HAL 100 microscope, ZEISS, Germany).

### VSV-G pseudo-typed lentivirus–shRNA production and infection

The shRNA-expressing lentivirus was purchased from National RNAi Core Facility located at the Institute of Molecular Biology/Genomic Research Center, Academia Sinica. Individual clones were identified by their unique TRC number: shLuc TRCN0000072246 for vector control targeted to luciferase; shATG5 (394) TRCN0000330394 (responding sequence: CCTGAACAGAATCATCCTTAA) and shATG5 (474) TRCN0000151474 (responding sequence: CCTTTCATTCAGAAGCTGTTT) targeted to ATG5. The detailed steps of lentivirus infection have been previously described.^17^

### Knockdown of CD133 expression using siRNA

A549/A400 cells at a density of 1 × 10^5^ were seeded in a 35 mm dish. After incubation for 16 h, the 30 pmol control siRNA and CD133-targeting siRNA (Sigma, siCD133#1 PROM1_Hs01_00100511; siCD133#2 PROM1_Hs01_00100514) were transfected using Lipofectamine RNAiMAX reagent (Invitrogen, Carlsbad, CA, USA). After transfection for 4 h, the transfection medium was replaced by fresh medium and incubated for an additional 72 h. The cells were harvested using trypsin/EDTA and reseeded for further analysis.

### Co-immunoprecipitation (co-IP)

A549/A400 cells (6 × 10^5^ cells) were seeded in a 100 mm dish containing 10 mL of culture medium. After 16 h incubation, the cells were treated with GMI and chloroquine for 24 h. Twenty microlitres of Protein A/G agarose (20422, ThermoFisher, USA) was added into 1 mg total protein for pre-clearing and rotated at 4 °C for 2 h. After centrifugation at 1000 RCF at 4 °C for 2 min, the supernatant was collected and then 2 μg anti-CD133 antibodies (#86781, Cell Signaling, Danvers, MA, USA) or anti-LC3B antibodies was added (#3868, Cell Signaling, Danvers, MA, USA) and rotated at 4 °C overnight. Fifty microlitres of Protein A/G was added and rotated at 4 °C for 1 h. After washing for three times, 25 μL sample buffer was added, boiled at 95 °C for 5 min and then spun down. The supernatant was analysed by the Western blot assay.

### Xenograft tumour model

All animal experimentation procedures were conducted according to the Affidavit of Approval of Animal Use Protocol, Chung Shan Medical University Experimental Animal Center, Taichung (Approval Number 2167). Male nude mice (BALB/c nu/nu mice) 5–6 weeks old and weighing 18–22 g were used for in vivo tumour growth assay. Mice were housed under pathogen-free conditions with a 12-h light/12-h dark cycle and fed an autoclaved diet with ad libitum access to standard rodent chow (LabDiet, 5001). The mice were subcutaneously injected with 5 × 10^6^ A549/A400 cells (100 μL) plus 100 μL of Matrigel (BD Biosciences, 354234) to establish tumour xenografts. Ten animals were then randomly divided into two groups consisting of five animals each. At 7 days after cell implantation, the mice in the Phosphate Buffered Saline (PBS) and GMI groups were treated with 100 μL of PBS and 160 μg of GMI (diluted in 100 μL of PBS, according to our previous study) by gavage once every morning until 1 day before mice were sacrificed. During experiment, health statuses of mice were monitored twice daily. No adverse events were observed. Tumour sizes were measured every 3 or 4 days after 27 days of cell injection, and tumour volume was calculated by the formula 0.5 × larger diameter (mm) × small diameter (mm)^2^. Mice were sacrificed via CO_2_ asphyxiation after 69 days of cell injection, and the tumour weights were measured by microbalance and analysed by immunohistochemistry (IHC).

### Statistical analysis

One-sample *t* test by Predictive Analytics SoftWare (PASW) Statistics 18 was used to conduct the statistical comparisons between two groups. *P* values of <0.05 were considered significant. Data are presented as mean ± SD.

## Results

### GMI inhibits cell viability in A549/A400 pemetrexed-resistant lung cancer cells

**Fig. 1:**
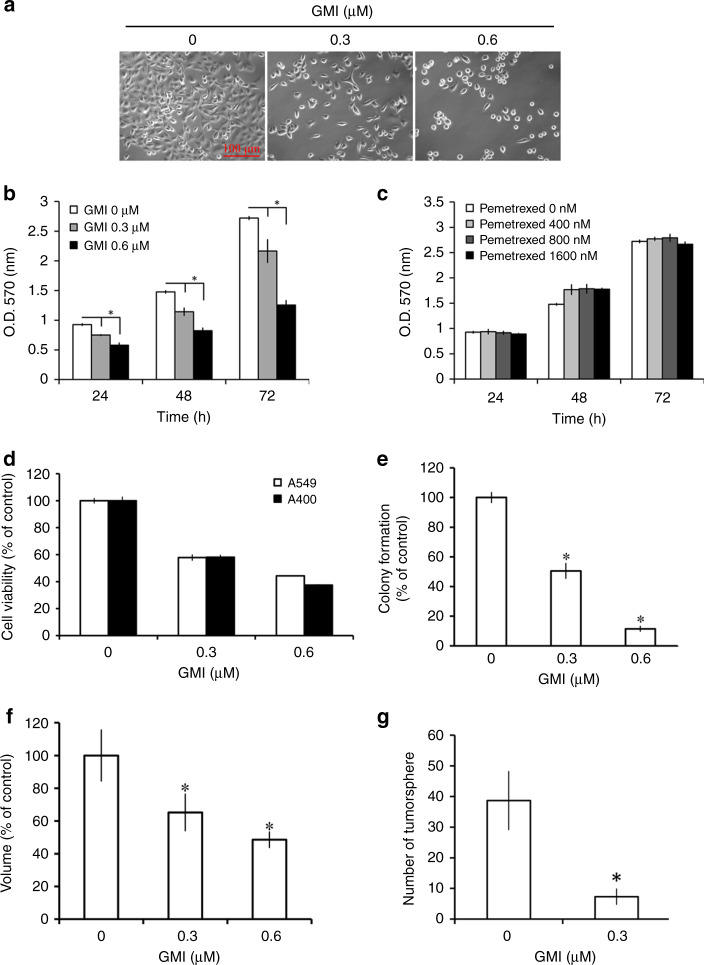
Effects of GMI and pemetrexed on cell viability in A549/A400 pemetrexed-resistant lung cancer cells. **a** After GMI (0, 0.3, and 0.6 μM) treatment for 48 h, the morphology of A549/A400 cells was investigated under an inverted microscope. Scale bar indicates 100 μm. **b** A549/A400 cells (2 × 10^3^ cells/well of 96-well plate) were treated with various concentrations of GMI (0, 0.3, and 0.6 μM) for 24, 48, and 72 h. Cell viability was analysed by MTT assay. **c** After treatment of pemetrexed (0, 400, 800, and 1600 nM) for an indicated time, MTT assay was performed to investigate the cell viability. **d** A549 and A549/A400 cells (2 × 10^3^ cells/well of 96-well plate) were treated with GMI (0, 0.3, and 0.6 μM) for 48 h. Cell viability was analysed by MTT assay. **e** A549/A400 cells (2 × 10^2^ cells/well of six-well dish) were treated with various concentrations of GMI (0, 0.3, and 0.6 μM). After treatment for 24 h, the original medium was replaced with fresh medium, and the cells were incubated for 14 days for colony development. The number of colonies was counted under a dissecting microscope. The number of cells in each colony had to be larger than 50. Data show the relative colony number, and the number of cells without treatment was set at 100%. **f** A549/A400 cells (1 × 10^3^ cells/well of 96-well dish) were seeded onto ultra-low attachment 96-well plates. After 96 h incubation for spheroid formation, GMI (0, 0.3, and 0.6 μM)-containing medium was added to the well, and the spheroids were incubated for 7 days. Spheroids were investigated under an inverted microscope. The volumes of spheroids were determined by the formula 0.5 × larger diameter × small diameter^2^. Data show the relative spheroid volume, and the volume of spheroid without treatment was set at 100%. **g** A549/A400 cells (5 × 10^3^ cells/well of a six-well plate) cultured in the sphere formation medium with or without 0.3 μM GMI. After 14 days, the spheres were investigated and counted under an inverted microscope. The symbol ‘*’ indicates *P* < 0.05.

The cell morphology and density of A549/A400 pemetrexed-resistant lung cancer cells were investigated under a microscope. Cell shrinkage and lower density were induced by GMI in A549/A400 cells (Fig. 1a). MTT assay showed that GMI inhibited the viability of A549/A400 cells in a dose- and time-dependent manner (Fig. 1b). As shown in Fig. 1c, it revealed a high resistance to pemetrexed in A549/A400 cells. The effects of GMI on parental A549 and A549/A400 are similar (Fig. 1d). Furthermore, several assays were performed to investigate the long-term effect of GMI on the survival of A549/A400 cells. Results of clonogenic assay showed that GMI significantly decreased the colony-formation ability of A549/A400 cells (Fig. 1e and Supplementary Fig. S1A). Three-dimensional tumour spheroid assay is an efficient model for investigating drug effects in vitro.^27^ As shown in Supplementary Fig. S1B, A549/A400 cells formed spheroids in the ultra-low attachment 96-well plate. GMI inhibited the spheroid size in a dose-dependent manner (Fig. 1f). Cancer stem cell sphere assay was performed to analyse the effect of GMI on the survival of cancer stem cells. GMI inhibited cancer stem cell sphere formation, suggesting that GMI abolished the survival of A549/A400 cells (Supplementary Fig. S1C and Fig. 1g). These results demonstrate that GMI inhibits viability of adherent and spheres derived from A549/A400 cells.

### GMI induces autophagy in A549/A400 cells

**Fig. 2:**
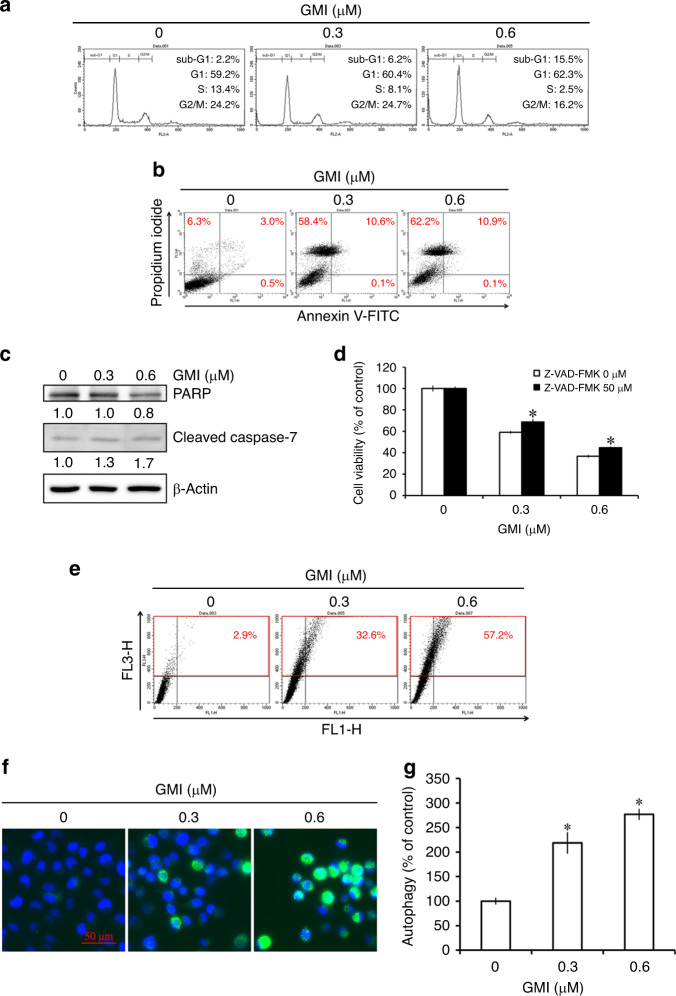
Effect of GMI on cell cycle distribution and induction of apoptosis and autophagy in A549/A400 cells. **a** A549/A400 cells (2 × 10^5^ cells of 60 mm dish) were treated with GMI (0, 0.3, and 0.6 μM) for 48 h. After PI staining, cells were analysed by flow cytometry. **b** After treatment with GMI (0, 0.3, and 0.6 μM) for 48 h, A549/A400 cells (2 × 10^5^ cells of 60 mm dish) were stained with annexin V-FITC/PI and analysed by flow cytometry. **c** A549/A400 cells (2 × 10^5^ cells of 60 mm dish) were treated with GMI for 48 h. Equal amounts of total cell lysates were analysed by Western blot assay. β-actin served as a loading control. **d** After GMI (0, 0.3, and 0.6 μM) and Z-VAD-FMK (0 and 50 μM) treatment for 48 h, MTT assay was performed to investigate the cell viability. **e** A549/A400 cells (2 × 10^5^ cells of 60 mm dish) treated with GMI (0, 0.3, and 0.6 μM) for 48 h were stained with acridine orange and analysed by flow cytometry. The upper right and upper left quadrants were quantified as AVO-positive cells. **f** After treatment of GMI for 48 h, A549/A400 cells (2 × 10^3^ cells/well of 96-well dish) were stained with CYTO-ID autophagy detection kit 2.0. The stained cells were investigated under a fluorescence microscope. Scale bar indicates 50 μm. **g** Fluorescence-activated A549/A400 cells were analysed with a fluorescence microplate reader. Results show the relative change in fluorescence strength, and the fluorescence strength of A549/A400 cells without treatment was set at 100%. The symbol ‘*’ indicates *P* < 0.05.

**Fig. 3:**
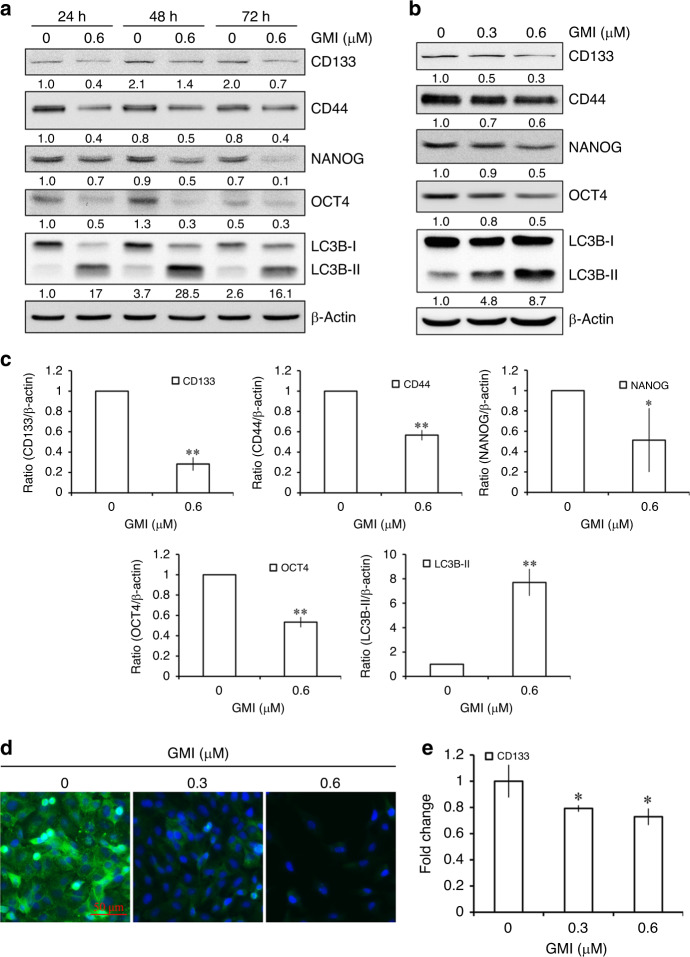
Effect of GMI on stemness genes in A549/A400 cells. **a** A549/A400 cells (2 × 10^5^ cells of 60 mm dish) were treated with GMI for indicated times. Equal amounts of total cell lysates were analysed by Western blot assay. β-actin served as a loading control. **b** CD133, CD44, NANOG, OCT4, LC3B, and β-actin were determined by Western blot after A549/A400 cells (2 × 10^5^ cells of 60 mm dish) treated with GMI (0, 0.3, and 0.6 μM) for 72 h. **c** Statistical analysis of Western blots. The band intensities of CD133, CD44, NANOG, OCT4, and LC3B-II were quantified using software ImageJ and standardised by β-actin. The ratio of cells without GMI treatment was set at 1. The symbols ‘*’ and ‘**’ indicate *P* < 0.05 and *P* < 0.001, respectively. **d** A549/A400 cells (2 × 10^5^ cells of 60 mm dish) were treated with GMI (0, 0.3, and 0.6 μM) for 72 h and then stained with anti-CD133 antibody. Scale bar indicates 50 μm. **e** Total RNA was collected from A549/A400 cells (2 × 10^5^ cells of 60 mm dish) after treating with GMI (0, 0.3, and 0.6 μM) for 72 h. RT-qPCR was performed to analyse the mRNA expression of CD133. The symbol ‘*’ indicates *P* < 0.05.

Flow cytometry was used to analyse the cell cycle distribution to elucidate the mechanism of cell death induced by GMI. GMI treatment caused a 10% increase in the sub-G1 population and a 10% decrease in the S phase population in A549/A400 cells (Fig. 2a). Annexin V/propidium iodide staining assay was used to analyse apoptotic cell death. As shown in Fig. 2b, GMI induced about 10% apoptosis in A549/A400 cells. GMI markedly increased the annexin V (−)/propidium iodide (+) population in A549/A400 cells (Fig. 2b). GMI partially decreased PARP expression and increased cleaved caspase-7 expression in A549/A400 cells (Fig. 2c). No cleaved PARP were observed after GMI treatment. Compared with GMI treatment, pan-caspase inhibitor Z-VAD-FMK increased 9% cell viability in A549/A400 cells (Fig. 2d). These results suggested that GMI predominantly induces non-apoptotic cell death in A549/A400 cells. In our previous studies, autophagy is the major cytotoxic pathway of GMI to kill cancer cells.^17,20^ Acridine orange staining assay was performed to detect the development of AVOs. GMI increased the development of AVOs in A549/A400 cells. At 0.3 and 0.6 μM concentrations of GMI, 32% and 57% of cells underwent autophagy, respectively (Fig. 2e). Furthermore, CYTO*-*ID Autophagy Detection Kit 2.0 was used to investigate autophagosome formation. GMI induced autophagosome formation in A549/A400 cells (Fig. 2f). Fluorescence intensity was detected with a fluorescence plate reader. As shown in Fig. 2g, GMI increased the autophagosome in A549/A400 cells in a dose-dependent manner. Furthermore, GMI significantly increased LC3-II expression in A549/A400 cells (Fig. 3a, b). These data provided the evidence that GMI induces autophagic cell death in A549/A400 cells.

### GMI inhibits lung cancer stemness genes in A549/A400 cells

As shown in Fig. 2e, GMI repressed the cancer stem cell sphere formation in A549/A400 cells. This result prompted us to examine whether GMI affects the expression of stemness genes of lung cancer in A549/A400 cells. CD133 and CD44 are lung cancer stem cell markers. Results of Western blot assay showed that 0.6 μM GMI reduced the protein expression of CD133 and CD44 after 24, 48, and 72 h treatment. Furthermore, GMI inhibited the expression of stemness-related transcriptional factors NANOG and OCT4 (Fig. 3a). A549 and CaLu-1 NSCLC cells were used to investigate the effect of CD133 inhibition of GMI. GMI inhibited CD133 expression in A549, but not in CaLu-1 cells (Supplementary Fig. S2A). After 72-h treatment, GMI repressed the expression of CD133, CD44, NANOG, and OCT4 in a dose-dependent manner (Fig. 3b). Statistical analysis of band intensity revealed that GMI significantly inhibited the protein expression of CD133, CD44, NANOG, and OCT4 (Fig. 3c). In addition, immunocytochemistry was performed and it showed the reduced expression of CD133 after GMI treatment (Fig. 3d). Furthermore, GMI reduced the mRNA expression of CD133 in A549/A400 cells (Fig. 3e). These results demonstrate that GMI lowers cancer stemness in A549/A400 cells.

### GMI induces CD133 protein degradation via autophagy

**Fig. 4:**
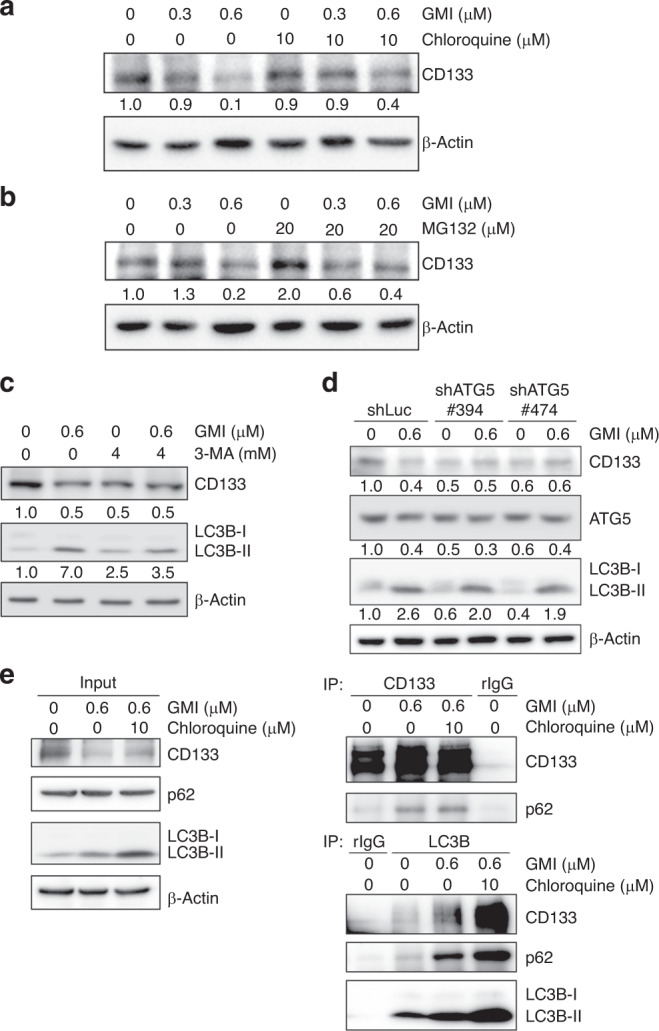
Effects of proteasome and autophagy on GMI-inhibited CD133 expression. **a** Total cell lysates of A549/A400 cells (2 × 10^5^ cells of 60 mm dish) after co-treating with GMI and chloroquine for 24 h were analysed by Western blot assay. **b** A549/A400 cells (2 × 10^5^ cells of 60 mm dish) were treated with MG132 for 6 h after treating with GMI for 18 h. Western blot assay was performed to detect the expression of CD133. **c** After pre-treating with 3-MA, A549/A400 cells (2 × 10^5^ cells of 60 mm dish) were treated with GMI for 24 h. The total protein was analysed by Western blot assay. **d** Total protein from A549/A400 shLuc and A549/A400 shATG5 cells was analysed by Western blot and detected by CD133, ATG5, LC3B, and β-actin antibodies. β-actin served as a loading control. Software ImageJ was used to quantify the band intensities of CD133, LC3B-II, and ATG5. Data shown are the relative expression standardised by the β-actin protein level. The ratio without treatment in A549/A400 or A549/A400 shLuc cells was set at 1. **e** After co-treating with GMI and chloroquine for 24 h, total cell lysates of A549/A400 cells (6 × 10^5^ cells of 100 mm dish) were used to investigate the interaction between CD133 and autophagosome protein (p62 and LC3B) by co-IP.

As displayed in Fig. 3, GMI inhibited the mRNA and protein expression of CD133. However, compared with untreated cells, GMI only decreased about 20% CD133 mRNA expression. This result suggests that GMI suppresses CD133 expression not only via transcriptional regulation. To elucidate the role of protein degradation in CD133 downregulation by GMI, we blocked lysosome- and proteasome-mediated protein degradation by using chloroquine and MG132, respectively. As shown in Fig. 4a, chloroquine partially rescued the GMI-mediated CD133 suppression. MG132 also partially reversed CD133 inhibition by GMI (Fig. 4b). However, CD133 expression was markedly increased after treatment with MG132 alone (Fig. 4b), suggesting that the reversal effect of MG132 is caused by blunting the intrinsic CD133 protein degradation. Autophagy is important in lysosomal protein degradation. To assess the role of autophagy in GMI-induced CD133 protein degradation, we inhibited autophagy by using autophagy inhibitor 3-MA and ATG5 shRNA. 3-MA inhibited LC3-II increase and CD133 decrease by GMI (Fig. 4c). ATG5 silencing mitigated the GMI-mediated LC3-II upregulation and CD133 downregulation (Fig. 4d). To further clarify the relationship between CD133 and autophagy, co-immunoprecipitation (co-IP) was performed to investigate whether CD133 is captured by the autophagosome. As shown in Fig. 4e (right upper panel), p62, an autophagy adaptor, was co-immunoprecipitated with CD133 from GMI-treated A549/A400 cells. LC3-II interacts with autophagy adaptors lead cargo to be engulfed by autophagosome.^28^ LC3B antibody used in this study shows higher affinity to LC3B-II than LC3B-I. Therefore, co-IP was also performed with LC3B antibody. After GMI treatment, CD133 and p62 were co-immunoprecipitated with LC3B (Fig. 4e, right lower panel). The interactions were increased after chloroquine treatment because the lysosomal protein degradation was blocked (Fig. 4e, right panel). These results demonstrate that GMI in part reduces CD133 expression via autophagic-lysosomal protein degradation.

### CD133 knockdown decreases cell survival in A549/A400 cells

**Fig. 5:**
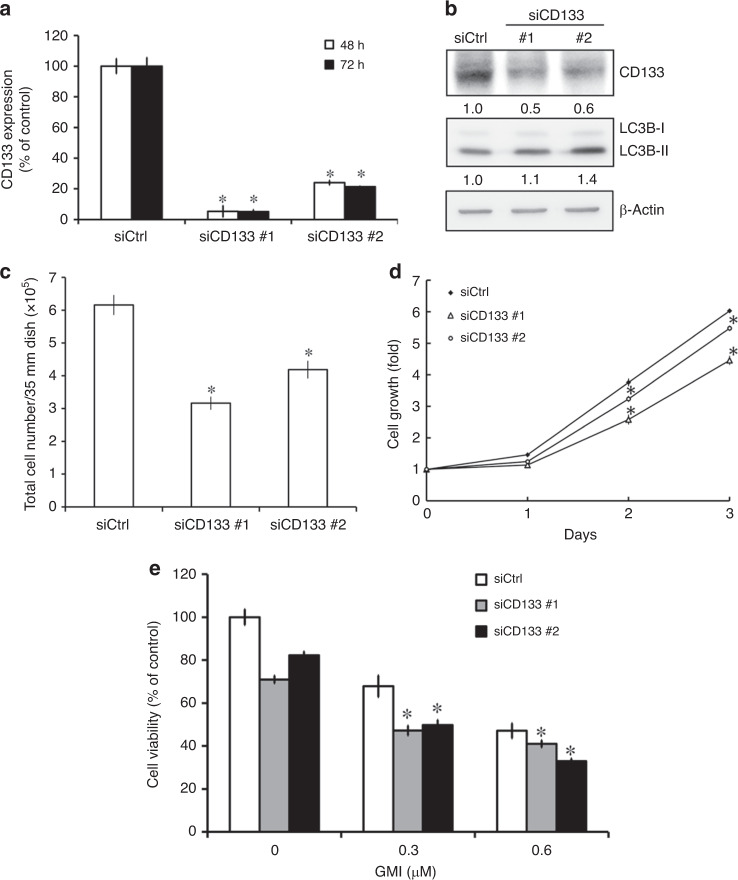
Effect of CD133 knockdown on cell survival and proliferation in A549/A400 cells. **a** A549/A400 cells (1 × 10^5^ cells of 35 mm dish) were transfected with control (Ctrl) siRNA and CD133 siRNA for 48 and 72 h. RT-qPCR was performed to analyse the mRNA expression of CD133. **b** A549/A400 cells (1 × 10^5^ cells of 35 mm dish) were transfected with control (Ctrl) siRNA and CD133 siRNA for 72 h. Equal amounts of total cell lysates of A549/A400 were analysed on Western blot, and the antibodies of CD133, LC3B, and β-actin were used to detect the expression of CD133, LC3B, and β-actin, respectively. The band intensities of CD133, LC3B, and β-actin were quantified using software ImageJ. Data shown are the relative expression standardised by the β-actin protein level. The ratio without treatment in A549/A400 siCtrl cells was set at 1. **c** After siRNA transfection for 72 h, A549/A400 cells were harvested by trypsin, stained with trypan blue, and counted under an inverted microscope. The symbol ‘*’ indicates *P* < 0.05. **d** After siRNA transfection for 72 h, A549/A400 siCtrl cells and A549/A400 siCD133 cells were harvested and seeded in 96-well dish at a density of 2 × 10^3^ cells per well. MTT assay was performed to analyse cell proliferation. The symbol ‘*’ indicates *P* < 0.05 for the siCD133 group when compared with the siCtrl group at the same time-point. **e** After siRNA transfection for 72 h, A549/A400 siCtrl cells and A549/A400 siCD133 cells were harvested and seeded in 96-well dish at a density of 2 × 10^3^ cells per well. After GMI treatment for 48 h, MTT assay was performed to analyse cell proliferation. The symbol ‘*’ indicates *P* < 0.05 for the GMI-treated siCD133 group when compared with the GMI or siCD133 alone group.

To investigate the role of CD133 in cancer cell survival, we established CD133 knockdown A549/A400 cells by using CD133-targeting siRNA. The different silencing effects of two siRNAs against CD133 were investigated in A549/A400 cells (Fig. 5a, b). CD133 silencing slightly increased LC3-II expression (Fig. 5b). As shown in Fig. 5c, the cell numbers were reduced significantly by siCD133 compared with siCtrl. Furthermore, CD133 knockdown significantly inhibited the proliferation of A549/A400 cells (Fig. 5d). The inhibiting effect of GMI on cell viability was enhanced in CD133-silencing A549/A400 cells (Fig. 5e). These results demonstrate that CD133 plays a role in cell growth in A549/A400 cells.

### GMI suppresses the tumour growth and CD133 expression in an A549/A400 xenograft tumour model

**Fig. 6:**
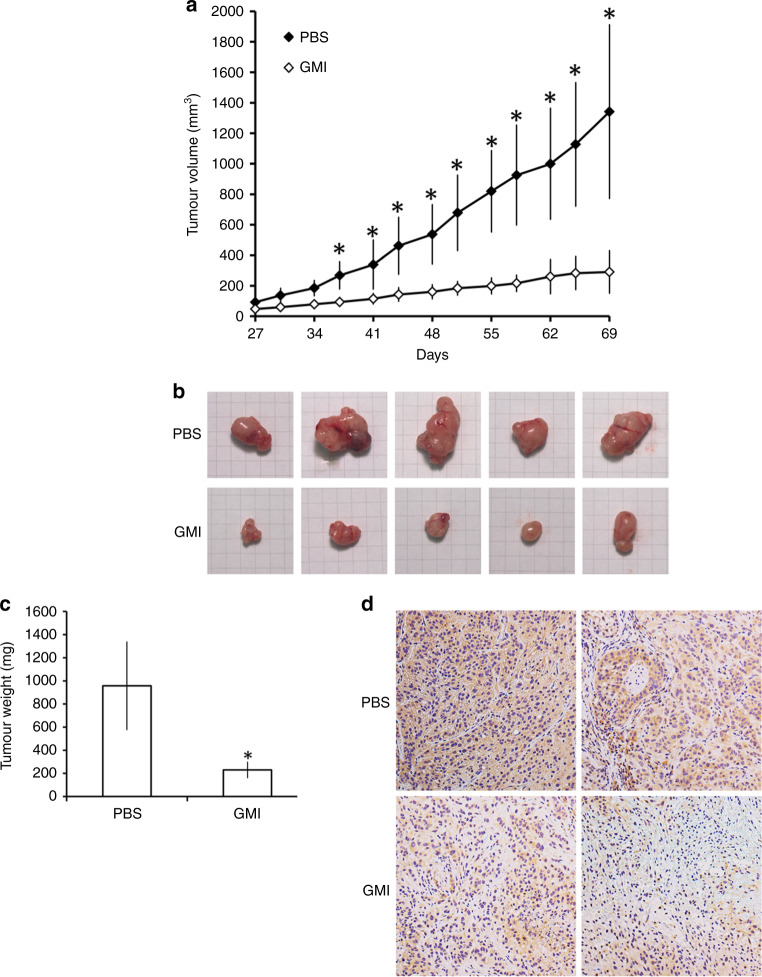
Effect of GMI on A549/A400 cell xenograft tumour growth in nude mice. **a** A549/A400 cells (approximately 5 × 10^6^) were injected subcutaneously into each left flank of nude mice to initiate tumour growth. At 7 days after implanting the cells, the PBS group started to receive sterilised PBS, whereas the mice in GMI group received GMI protein (160 μg/mouse, *N* = 5, respectively). PBS and GMI proteins were administered to mice by gavage once every day. At 27 days after cell transplantation, tumour sizes were measured every 3 or 4 days and the tumour volume was calculated. The symbol ‘*’ indicates *P* < 0.05 for the tumour volume of the PBS group when compared with the tumour volume of the GMI group at the same time-point. **b** Tumour images of the PBS and GMI groups. **c** Tumour weights were measured after the mice were sacrificed at day 69. The symbol ‘*’ indicates *P* < 0.05. **d** IHC staining was performed to investigate CD133 expression in tumours of the PBS and GMI groups.

To investigate the effect of GMI on anti-pemetrexed-resistant lung cancer, we performed an in vivo study using a nude mouse xenograft model subcutaneously inoculated with A549/A400 cells. As shown in Fig. 6a, the average tumour volume in the GMI treatment group was significantly lower than that in the control group at day 37. The mice were sacrificed at day 69, and the tumour size and weight in the GMI group were statistically lower than those in the PBS group (Fig. 6b, c). CD133 expression in the tumour was analysed by IHC. As shown in Fig. 6d, GMI decreased the CD133 expression in the xenograft tumour. These data demonstrate that GMI inhibits A549/A400 tumour growth and CD133 expression in vivo via oral administration.

## Discussion

High levels of thymidylate synthase expression lead to pemetrexed resistance in lung cancer.^15,16^ Knockdown of thymidylate synthase reverses the resistance of pemetrexed.^15,16^ We found that GMI inhibited the expression of thymidylate synthase in parental A549 and A549/A400 cells (Supplementary Fig. S2A). However, GMI partially increased the pemetrexed sensitivity in A549, but not in A549/A400 cells (Supplementary Fig. S2B, C). Regardless of the effect of GMI on pemetrexed, thymidylate synthase inhibition provides an alternative route for GMI to suppress lung cancer proliferation. In our previous study, GMI induces autophagic cell death in lung cancer cells.^17^ In the present study, we found that GMI induced autophagy markedly but not apoptosis, suggesting that GMI induces the cytotoxicity in the pemetrexed-resistant lung cancer cells via autophagy.

Co-expression of NANOG and OCT4 reportedly enhances the malignancy of lung adenocarcinoma by enhancing the properties of cancer stem cells.^29^ Inhibition of the IL-6/Stat3 signalling pathway by GMI abolishes cancer stemness in oral carcinoma stem cells.^22^ In the present study, GMI decreased the expression of stemness*-*related transcriptional factors NANOG and OCT4 and lung cancer stem cell markers CD133 and CD44. However, GMI only decreased 20% expression of CD133 mRNA, suggesting that GMI does not predominantly suppress CD133 expression by transcriptional regulation of NANOG and OCT4 but protein degradation. Interestingly, CD133 reportedly regulates the expression of NANOG and OCT4, suggesting that CD133 inhibition induces the GMI-mediated reduction in NANOG and OCT4.^10^ Regardless of the regulation on CD133, inhibition of NANOG and OCT4 suggested the potential of GMI in restraining cancer stem cells.

Proteasome and autophagy are major protein degradation systems in cells that control protein quality.^30^ Ubiquitin is a common degron of proteasomal and autophagic protein degradation.^31^ During autophagy, polyubiquitinated proteins interact with autophagic adapters, such as p62 and NBR1, and are engulfed in autophagosomes and degraded by lysosome.^32^ As shown in Fig. 4, the CD133 degradation induced by GMI was confirmed to be via the autophagy−lysosome pathway after using inhibitors of lysosome, proteasome, and autophagy. Furthermore, the results of co-IP assay demonstrated that CD133 was captured by autophagosome after GMI treatment. Interestingly, 3-MA and ATG5 shRNA decreased CD133 protein expression (Fig. 4c, d). These results are consistent with the findings of a recent study. Yang et al.^33^ found that autophagy inhibition by 3-MA and Beclin-1 depletion reduces the protein expression of CD133 in adherent and tumorsphere, and the tumorsphere formation in cervical cancer cells. Autophagy has been reported as a mechanism of cancer stem cells maintenance, suggesting that autophagy regulates CD133 expression via altering the population of cancer stem cells.^34^ Further experiments are needed to investigate the mechanism of CD133 regulation by autophagy. These findings demonstrate that intrinsic autophagy is critical for the CD133 expression and cancer stem cell maintenance.

CD133 is a well-known marker of cancer stem cells; however, the function of CD133 in normal or cancer cells is beyond a stem cell biomarker.^9,35^ β-catenin is activated in CD133-positive dermal papilla cells and controls the growth of postnatal hair, suggesting that CD133 can induce β-catenin activation in cancer.^36^ In glioma cancer stem cells, the interaction of CD133 with p85 promotes tumorigenicity via activating the PI3K/Akt signalling pathway.^37^ CD133 regulates Src activity, and the CD133/Src axis plays an important role in stemness, EMT, and tumorigenicity in head and neck cancer cells.^10^ In glioblastoma stem cells, CD133 promotes cell proliferation and xenograft tumour growth.^38^ CD133 promotes the survival of hepatoma cells via activating autophagy.^39^ In our previous study, GMI inhibition of Akt activity leads to autophagy induction in lung cancer cells.^18^ Our recent publication has demonstrated that GMI induces apoptosis by inhibiting β-catenin expression.^21^ In the present study, CD133 silencing decreased the proliferation of A549/A400 cells (Fig. 5c, d). However, CD133 silencing only slightly activated autophagy, suggesting that CD133 does not participate in GMI-induced autophagy (Fig. 5b). Furthermore, GMI inhibited Src activity in A549/A400 cells (data not shown). Taken together, GMI-inhibited CD133 has a great potential to restrain tumour growth.

The cell surface CD133 is a cancer stem cell marker. However, cytoplasmic CD133 may also play an important role in the self-renewal capacity of cancer cells.^38^ Brescia et al.^38^ demonstrated that CD133 silencing decreases the proliferation and sphere formation in glioblastoma stem cells with plasma membrane CD133 localisation and cytoplasmic CD133 localisation. In the present study, Western blot assay and immunocytochemistry were performed to investigate the decrease in CD133 after GMI treatment (Fig. 3a, b, d). However, GMI did not alter the expression of surface CD133 in A549/A400 cells (Supplementary Fig. S3). These results suggest that GMI induces the protein degradation of cytoplasmic CD133. In our previous study, GMI induces autophagy via ER stress stimulation.^17^ This result suggests that GMI elicits CD133 inhibition at least partially via ER stress-mediated CD133 protein misfolding and following the autophagic degradation of CD133. Further experiments and studies are needed to prove the hypothesis.

In conclusion, GMI inhibits cell viability and CD133 expression via autophagy in pemetrexed-resistant lung cancer cells. CD133 inhibition decreases the cell survival of pemetrexed-resistant lung cancer cells. This study provides new insights into the regulatory effect of GMI on CD133 and the role of CD133 in drug-resistant cancer survival.

## Supplementary information

Supplementary Figures

## Data Availability

The datasets supporting the conclusions are included within the article.
