# Supplementary material for: CD133 inhibition via autophagic degradation in pemetrexed-resistant lung cancer cells by GMI, a fungal immunomodulatory protein from Ganoderma microsporum
Source: Br J Cancer. 2020 May 25;123(3):449–58. doi: 10.1038/s41416-020-0885-8 (PMC7403151; doi:10.1038/s41416-020-0885-8)
Supplement: Supplementary file 1 — Supplementary Figures [file 41416_2020_885_MOESM1_ESM.docx]

1. (B)


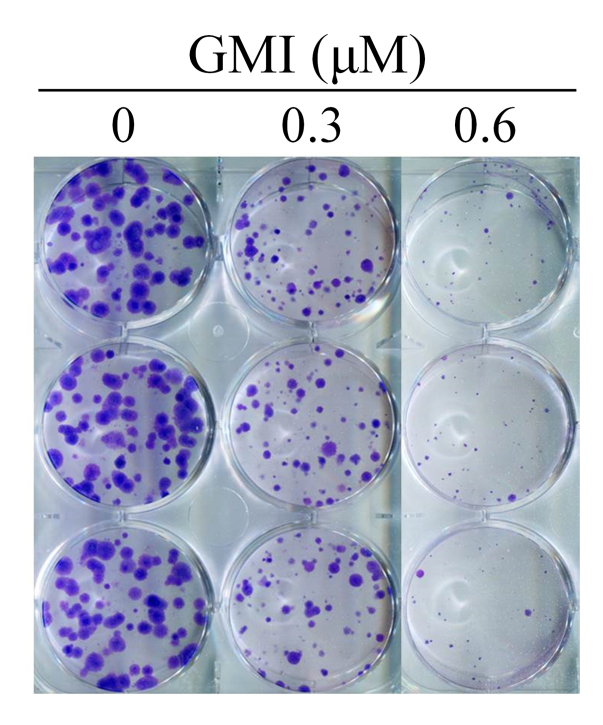

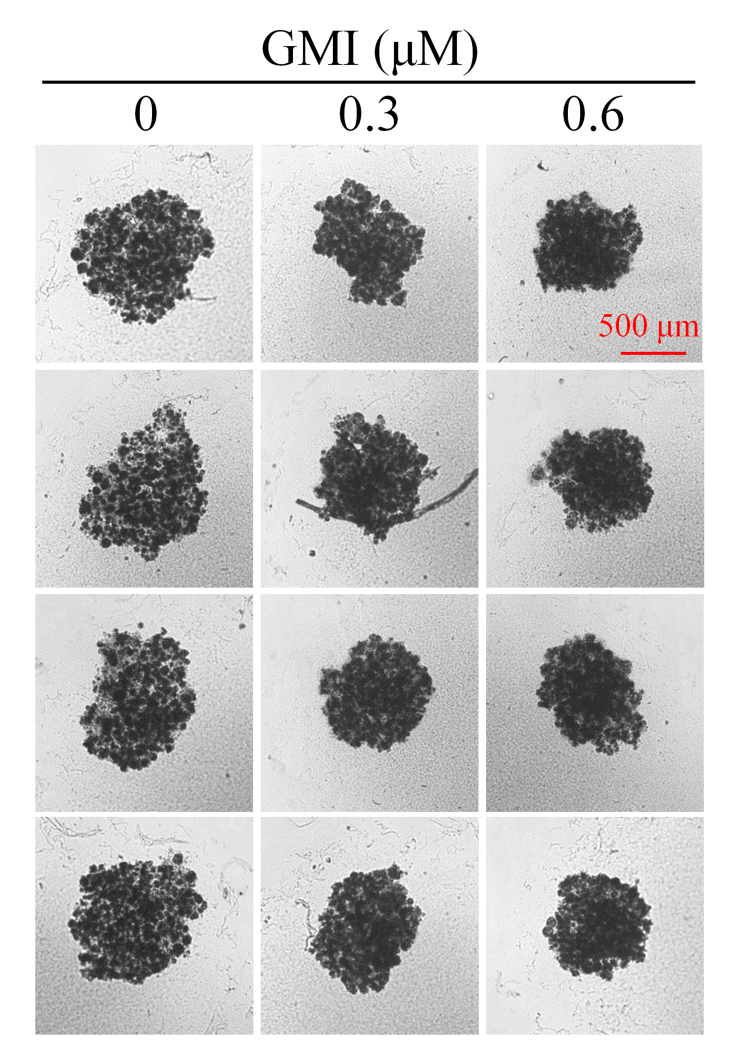


(C)


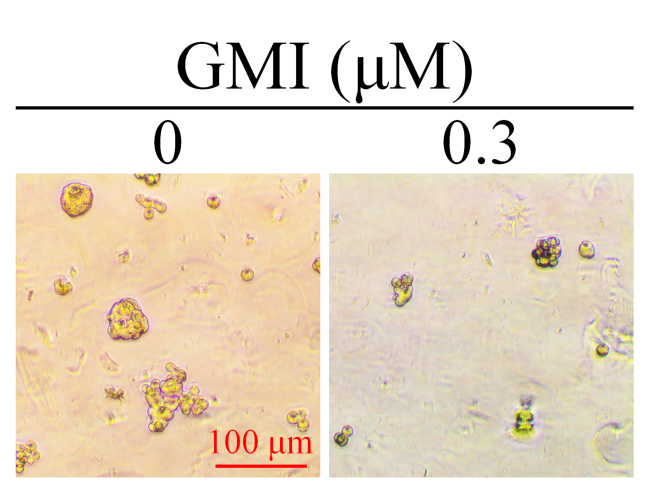


**Figure S1. GMI inhibits colony formation, tumor spheroid, and cancer stem cell sphere formation in A549/A400 cells**

(A) A549/A400 cells (2 × 10^2^ cells/well of 6-well dish) were treated with various concentrations of GMI (0, 0.3, and 0.6 μM). After treatment for 24 h, the original medium was replaced with fresh medium, and the cells were incubated for 14 days for colony development. (B) A549/A400 cells (1 × 10^3^ cells/well of 96-well dish) were seeded onto ultra-low attachment 96-well plates. After 96 h incubation for spheroid formation, GMI (0, 0.3, and 0.6 μM)-containing medium was added to the well, and the spheroids were incubated for 7 days. Scale bar indicates 500 μm. (C) A549/A400 cells (5 × 10^3^ cells/well of a 6-well plate) cultured in the sphere formation medium with or without 0.3 μM GMI. After 14 days, the spheres were investigated under an inverted microscope.

(A) (B)


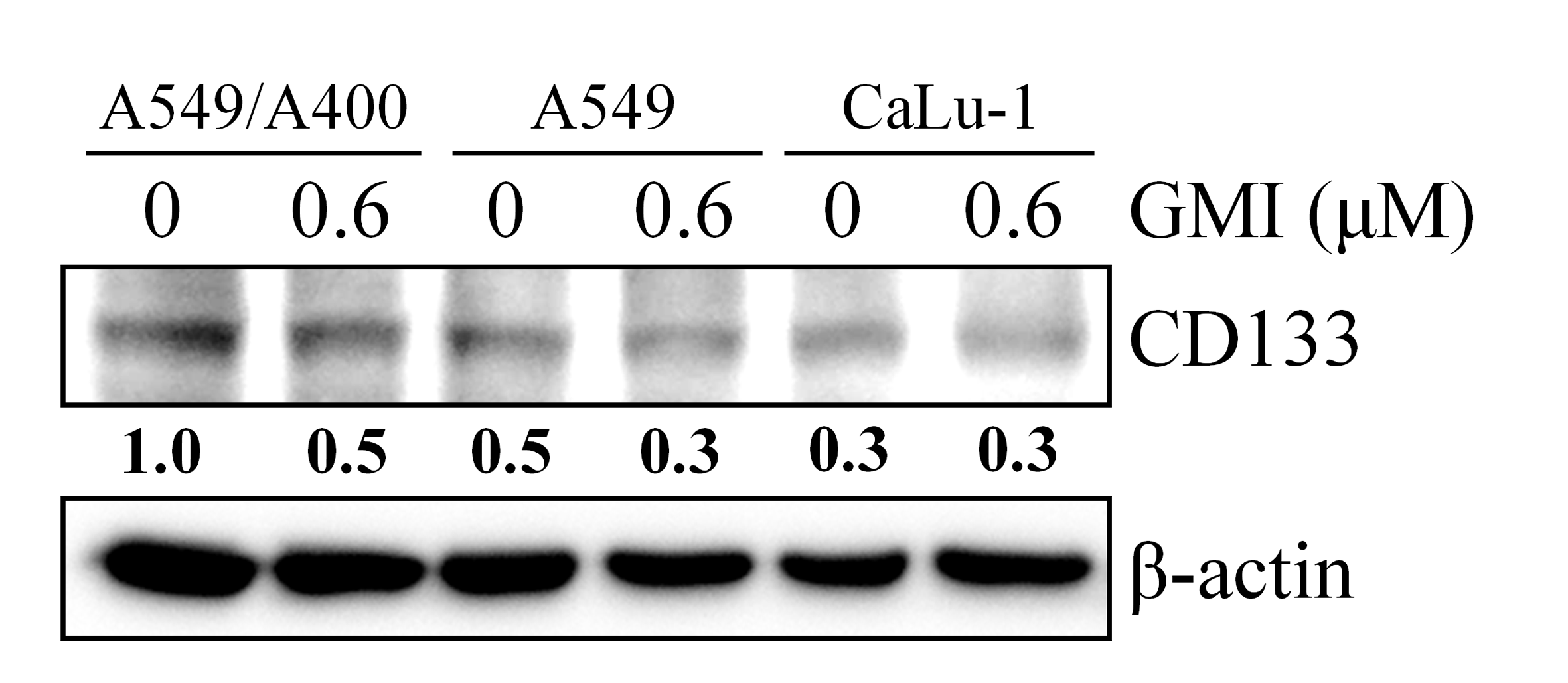

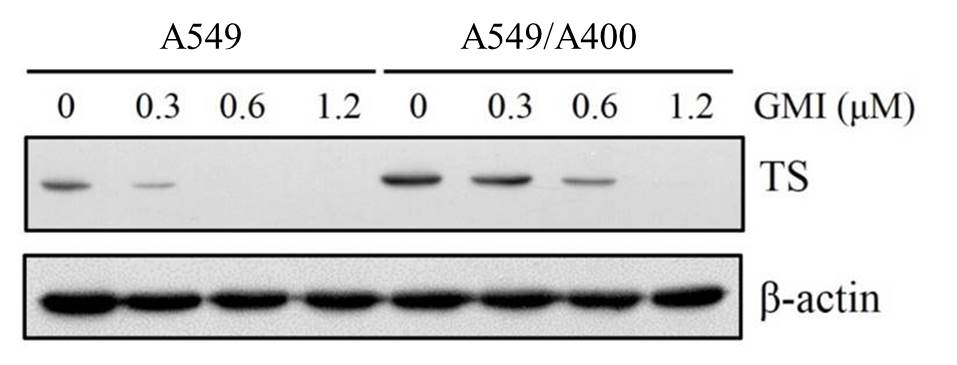


(C) A549 (D) A549/A400


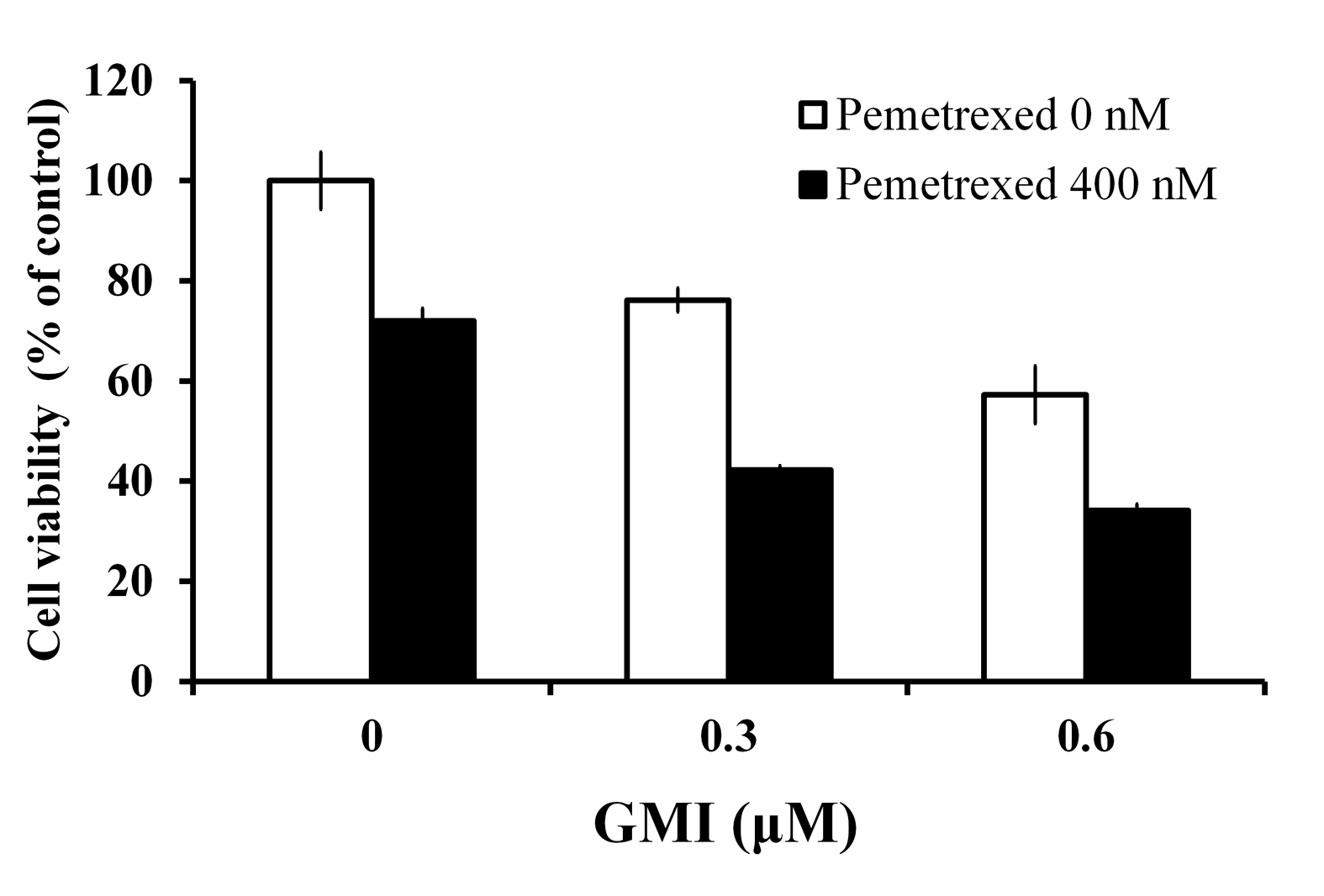

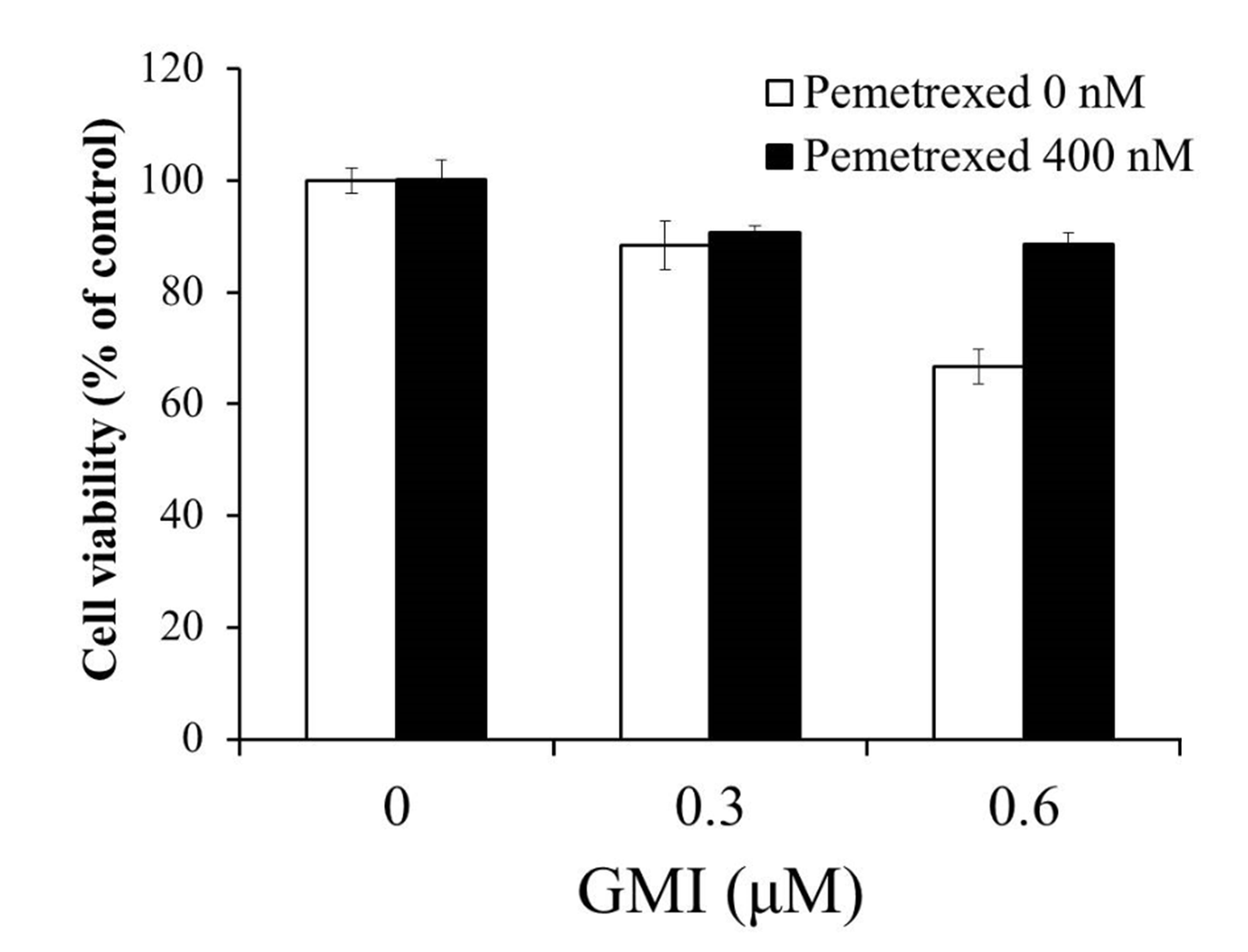


**Figure S2. Effect of GMI on protein expression of CD133 and thymidylate synthase (TS), and pemetrexed-inhibited cell viability**

(A) A549/A400, A549 and CaLu-1 cells (2 × 10^5^ cells of 60 mm dish) were treated with GMI for 24 h. CD133 expressions were analyzed by Western blot assay. (B) A549 and A549/A400 cells (2 × 10^5^ cells of 60 mm dish) were treated with GMI for 48 h. Equal amounts of total cell lysates were analyzed by Western blot assay. β-actin served as a loading control. (C) A549 and (D) A549/A400 cells (2 × 10^3^ cells/well of 96-well plate) were treated with various concentrations of GMI (0, 0.3, and 0.6 μM) for 72 h. Cell viability was analyzed by MTT assay.


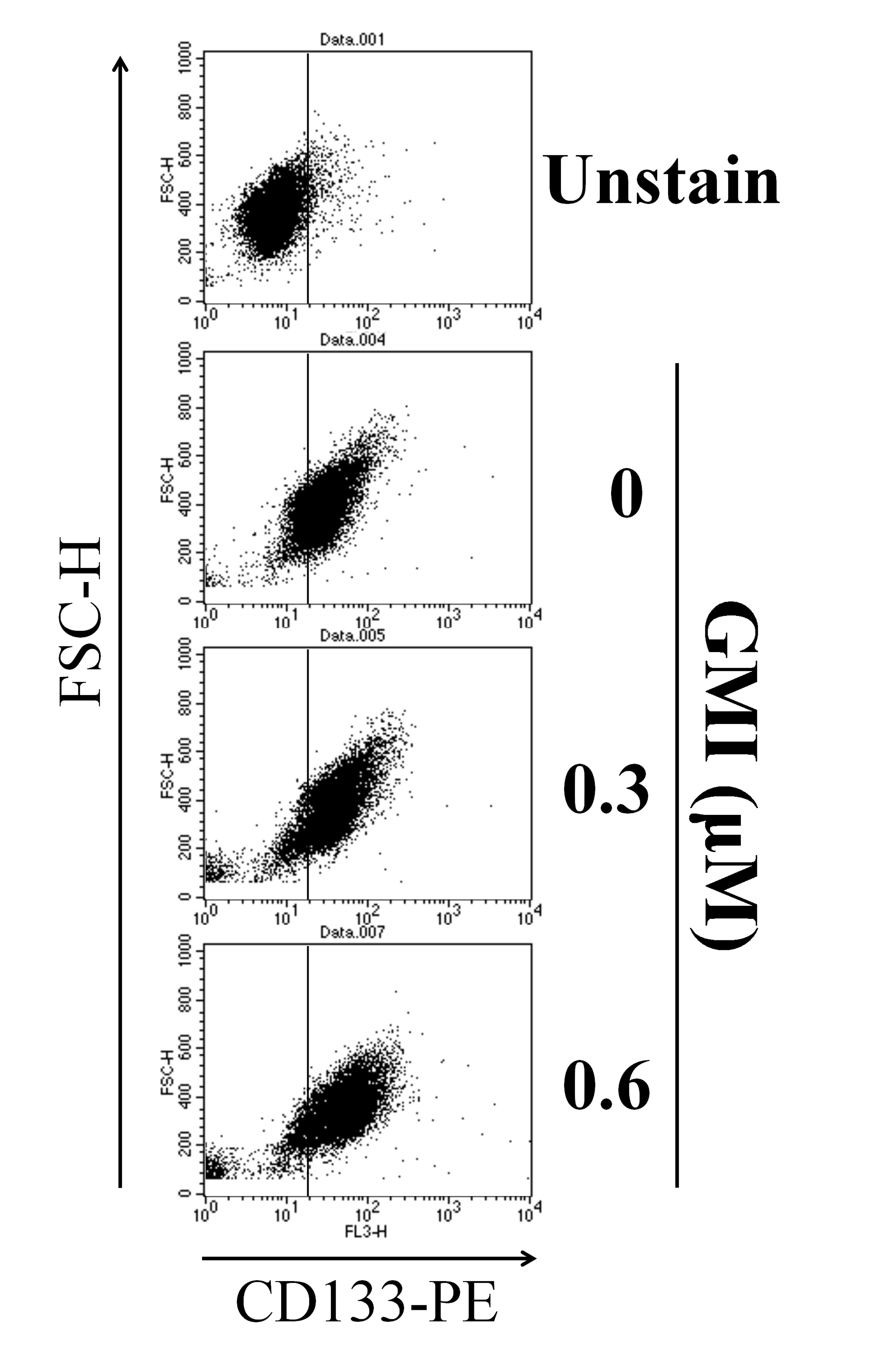


**Figure S3. Effect of GMI on the expression of surface CD133 in A549/A400 cells**

After GMI treatment for 72h, cells were collected by HyQTase cell detachment reagent (SV30030.01, Hyclone, USA) and stained with phycoerythrin (PE) conjugated anti-CD133 antibody (130-080-801, Miltenyi Biotec, USA). The unstained and stained cells were analyzed by flow cytometry.
